# Tuberculosis but not Immune Reconstitution Inflammatory Syndrome (IRIS) prevents early and late NK cell degranulation reconstitution in HIV/TB co-infected patients

**Published:** 2011-01-10

**Authors:** Polidy Pean, Eric Nerrienet, Yoann Madec, Laurence Borand, Sarin Chan, Olivier Marcy, Marcelo Fernandez, Françoise Barré-Sinoussi, Gianfranco Pancino, Daniel Scott-Algara

**Affiliations:** 1 Institut Pasteur in Cambodia Phnom Penh Cambodia; 2 Institut Pasteur, Paris, France; 3 Cambodian Health Committee, Phnom Penh, Cambodia

## Background

This study aims to evaluate the reconstitution of NK cell functions in HIV/TB co-infected patients developing IRIS versus those not having IRIS after TB and anti-retroviral treatment initiation.

## Methods

(i) 138 HIV+/TB+ patients enrolled in the CAMELIA trial (ANRS 1295-CIPRA/KH001-DAIDS-ES ID10425) in Cambodia and 36 HIV+ patients with CD4^+^ T cells < 200/mm^3^ included (ii) NK cells repertoire analysis by immuno-staining in whole blood and, (iii) NK cells degranulation activity and cytokine production by CD107a essay with and without stimulation. After starting of TB therapy in HIV+/TB+ patients, the time points were weeks 2, 8, 14 and 34. Half of the patients started HAART at week 2 and the other half at week 8. The time point of HIV+/TB- were week 0 and 8. The results obtained at baseline and following HAART are presented.

## Results

37/138 HIV+/TB+ patients developed IRIS. 33 IRIS, 67 non IRIS and 36 HIV+/TB- were available for CD107a degranulation and IFNγ production analysis and 32 IRIS, 78 non IRIS and 36 HIV+/TB- for NK cell repertoires analysis. At baseline, CD107a degranulation was lower in HIV+/TB+ (IRIS and non IRIS) than in HIV+/TB- (median 8.88 and 6.62 vs 12.81, p=0.008; p<0.0001). The IFNγ production was also lower in HIV+/TB+ (median 1.91 and 2.46 vs 6.55, p<0.001). From baseline to 6 weeks of HAART, CD107a degranulation in HIV+/TB+ (IRIS and non IRIS) were lower than 8 weeks treated HIV+/TB- (0.09 and 0.51 vs 7.53; p=0.04 and p=0.002, respectively), whereas the IFNγ production was not different (p>0.05). At baseline, CD69 positive NK cells in HIV+/TB+ (IRIS and non IRIS) was higher than HIV+/TB-. After 6/8 weeks of HAART, NK cells activation decreased and there were no difference in all groups while the expression of NKG2D, NKp30, and NKp46 among NK cells in non IRIS, but not in IRIS were higher than HIV+/TB- [(median +1.40 vs -0.95 , p=0.03) ; (median +1.39 vs -3.66, p=0.01) and (median -0.13 vs -3.53 , p=0.05) respectively]. Concerning levels of NK receptors, we observed several differences in particular NKG2C which was higher in IRIS patients compared to non IRIS patients at week 34.

## Conclusion

Co infection with Tuberculosis in HIV infected patients prevents NK cell degranulation reconstitution after TB and HAART treatment.

